# Consumer Expectations and Attitudes About Psychotherapy: Survey Study

**DOI:** 10.2196/38696

**Published:** 2023-06-08

**Authors:** Erin O'Callaghan, Heather Belanger, Steven Lucero, Shannon Boston, Mirene Winsberg

**Affiliations:** 1 Brightside Health Oakland, CA United States; 2 Department of Psychiatry and Behavioral Neurosciences University of South Florida Tampa, FL United States

**Keywords:** internet, survey, psychotherapy, telehealth, psychiatry, mental health

## Abstract

**Background:**

Although mental illness is common among adults in the United States, access to, and public perception of, mental health care continue to present as key barriers to care.

**Objective:**

Given the importance of attitudes toward, and perceptions of, mental health treatment in the successful access to mental health care and treatment of mental health issues, the primary goal of this survey study was to further investigate consumer perspectives of psychotherapy among adults in the United States; specifically, adding to the literature by investigating perceptions of both the general public and patients receiving telehealth. More specifically, the aims were to better understand openness to, and satisfaction with, therapy; perceptions, preferences, and expectations around therapy; and perceptions of psychotropic medication.

**Methods:**

An electronic survey was administered to current and former patients (those receiving psychotherapy) of Brightside, a nationwide telehealth company, as well as to the general public; both were convenience samples. Using the same survey questions, Brightside surveyed its own members (using Qualtrics; Qualtrics International Inc) and the general population (using SurveyMonkey’s Audience tool; Momentive). This survey included questions about basic participant demographics, as well as questions about current mental health treatment, perceptions about therapy, and therapists’ qualities.

**Results:**

A total of 714 people completed the survey. The data were fairly evenly split between those collected from Brightside patients (368/714, 51.5%) and those collected from the general public (346/714, 48.5%). Combining both samples, overall participation was 67.1% (479/714) women; 73.1% (522/714) White, 7.3% (52/714) Asian, 6.7% (48/714) African American, and 7.4% (53/714) Hispanic or Latinx; largely aged 25 to 34 years (255/714, 35.7%) or 35 to 44 years (187/714, 26.2%); from either the Mid-Atlantic (131/714, 18.3%) or South Atlantic (129/714, 18.1%) regions of the country; and most (402/714, 56.3%) earning annual salaries of US $30,000 to US $100,000. There were generally favorable perceptions of both psychotherapy and psychiatric medication. Selecting a therapist as well as cost and insurance are the common factors in therapy that are important to patients. The most commonly held perception of psychotherapy duration was *indefinite* (250/714, 35%). Very few (58/714, 8.1%) thought that therapy typically lasts 1 to 3 months. Most of the participants (414/714, 58%) thought that evidence-based practice was important.

**Conclusions:**

Public education is needed to increase awareness of the typical duration and cost of psychotherapy. There seem to be generally favorable perceptions of both psychotherapy and psychotropic medication. Selecting a therapist as well as cost and insurance are the common factors in therapy that are important to patients. Practitioners and those marketing their services might consider using their marketing campaigns to counter some of the more common falsely held beliefs.

## Introduction

### Background

Although mental illness is common among adults in the United States, access to, and public perception of, mental health care continue to present as key barriers to care. According to the National Institute of Mental Health, it is estimated that 1 in 5 adults in the United States—52.9 million people—experienced mental illness in 2020 alone [1]. The National Health Interview Survey conducted by the Centers for Disease Control and Prevention found that 19.2% of adults received mental health treatment in 2019, including 15.8% who had taken prescription medications for their mental health and 9.5% who received counseling or therapy from a mental health professional [2]. That said, estimates suggest that less than half of the people who need mental health services receive them, with access to psychotherapy particularly being limited [3]. Critically, it is well documented that the COVID-19 pandemic has exacerbated mental health issues across the country in recent years; for example, depression symptom prevalence was >3-fold higher during the COVID-19 pandemic than before [4].

Given the heightened need for mental health care, it is important to understand the barriers to seeking psychotherapy. The importance of patient attitudes and expectations of mental health treatment, particularly for success in psychotherapy, is well documented [5]. Research suggests that public perceptions of, and attitudes toward, therapy may mediate behavioral health care use rates. The literature has established a clear relationship between patients’ expectations and patient-rated therapeutic alliance [6], and therapeutic alliance may mediate the relationship between therapy-related expectancies and outcome [7]. Moreover, psychotherapy aligned with patient preferences tends to result in less attrition and greater clinical improvement [8,9]. Patient preferences, in turn, likely exert their influence via therapy expectations [10].

Many surveys of therapists’ beliefs, perceptions, and attitudes have been conducted, but far fewer have sought patient input as consumers of therapy services. Those surveys that have been directed at patients as consumers have focused on stigma [11], views on therapeutic alliance [12], views on evidence-based practice versus common-factor approaches [13], views on the relative importance of relational and scientific evidence for therapy received [13,14], and perceptions about provider credentials and referral sources [15]. Beyond these studies, little is understood about what patients value in psychotherapy, why potential patients pursue or do not pursue psychotherapy, and perceptions about psychotropic medications. Finally, to our knowledge, there are no surveys of consumer preferences from existing and potential samples using only telehealth.

### Objectives

Given the importance of attitudes toward, and perceptions of, mental health treatment in the successful access to mental health care and treatment of mental health issues, the primary goal of this survey study was to further investigate the consumer perspective of psychotherapy; specifically, adding to the literature by investigating perceptions of both the general public and patients receiving telehealth. Within this context, psychotherapy is defined as an evidence-based intervention provided by licensed mental health professionals during regularly scheduled sessions, which may include psychotropic medication prescribed during treatment. More specifically, the aims were to better understand the following aspects:

1. Openness to, and satisfaction with, therapy
2. Perceptions, preferences, and expectations around therapy
3. Perceptions of psychotropic medication

## Methods

### Participants

The participants represent a convenience sample recruited from the population of existing and former patients of Brightside, an internet-based national telemental health company. Additional participants were recruited from the general population to widen the sample and obtain a broader range of perspectives around attitudes to, and perceptions of, psychotherapy.

Brightside provides psychiatric services, including therapy and psychopharmacological treatment, via a web-based platform that leverages a measurement-based approach to track patient outcomes and offer clinical decision support to providers [16]. At Brightside, psychotherapy is based on the Unified Protocol (UP) [17], which is a transdiagnostic, evidence-based intervention rooted in cognitive behavioral therapy (CBT) approaches. Brightside patients receive treatment for a wide range of diagnoses across the range of emotional disorders. Of note, Brightside is not equipped to provide care for schizophrenia, psychotic disorders, or complex eating disorders, which is reflected in the convenience sample used. Brightside patients are also provided with self-care psychoeducational videos, either when they go through the intake process and choose not to sign up for treatment or when they cancel their treatment plans.

Those recruited from the general population were also selected as part of a convenience sample, and participation was limited to those residing in the United States, with gender balancing based on the most recent US census. The selection criteria for this sample were informed by SurveyMonkey’s Audience tool (Momentive), which allows researchers to access a public audience of >175 million SurveyMonkey users in >130 countries. Brightside researchers provided SurveyMonkey with the following desired demographic criteria for audience inclusion: the United States as country of residence, all genders, and participants aged 18 to 44 years.

### Measure

The survey used was developed for marketing and market analysis purposes. It included questions about basic demographics, as well as questions about current mental health treatment, perceptions about therapy, and therapists’ qualities. Many questions allowed respondents to choose *all options that apply* such that multiple responses per respondent were possible. Four variants of the survey were administered depending on the population it was administered to: (1) the general population and Brightside patients receiving self-care support, (2) Brightside patients receiving solely psychotherapy treatment, (3) Brightside patients receiving solely medication treatment, and (4) Brightside patients receiving both psychotherapy and medication treatments. Each survey contained 11 questions and differed only in the branching logic applied to question 1, which was intended to better define the characteristics of the population being surveyed. All questions administered in the survey can be found in Multimedia Appendix 1.

### Ethical Considerations, Privacy Policy, and Participation

This research was conducted in compliance with standards of research involving human participants. Anonymous, deidentified data were collected and analyzed for the purposes of better understanding perceptions of psychotherapy among a diverse group. Institutional review board (IRB) consent review was not obtained because our informed consent protocol allows for the secondary analysis of data without additional consent. Although not required, IRB approval was sought after survey completion; WCG IRB did not retrospectively determine exemption (because the research only involved a survey), but it provided us with a letter stating that had a review been requested before the survey administration, the study would likely have been found to be exempt from IRB review. As this survey originated as a marketing survey tool, the administering telemental health company received consent from users (existing patients) via a privacy policy or notice of privacy practice opt-in to receive marketing research materials. The terms of these agreements state that, occasionally, Brightside will engage in research to support ongoing medical and treatment insights. All published findings include only fully anonymized data, but certain research collaborators may require access to protected health information for the purposes of confidential data analysis. Furthermore, Brightside may use personal information and other information about patients to create deidentified and aggregated information, such as deidentified demographic information and deidentified location information. Deidentified information is not afforded the protections set forth by the Health Insurance Portability and Accountability Act (HIPAA) or other state privacy legislation because the information cannot be linked back to a specific individual after being deidentified.

Similarly, SurveyMonkey’s anonymous response collector allowed us to collect deidentified results from the general public respondents. As Brightside is the survey creator, its privacy policy or notice of privacy practice and its terms extend to all general public respondents. As an independent entity, SurveyMonkey records respondent IP addresses in backend logs, which are deleted after 13 months; all respondents agree to these terms before survey completion.

Monetary compensation was not provided for study participation in either the Brightside or general population groups.

### Procedure

The survey was administered by Brightside via email on September 22, 2021. Brightside surveyed its own members (using Qualtrics; Qualtrics International Inc) and the general population (using SurveyMonkey). The survey was left open for 2 days. Participants could choose to opt out and discontinue completing the survey at any time. The survey administered using SurveyMonkey had a 9% abandonment rate, whereas the survey administered using Qualtrics had a 12% abandonment rate.

### Analysis

Descriptive statistics are presented to summarize the results. The willingness to engage in psychotherapy (and perceptions thereof) were investigated among those who are currently engaged in mental health treatment as well as among those who are not. Subsequently, perceptions about psychotherapy were further summarized by those currently engaged in (1) pharmacotherapy only, (2) psychotherapy only, or (3) a combination of both. For questions about evidence-based therapy, those using Brightside services versus those not using them were compared. Comparisons between subgroups were made using chi-square analyses evaluated at *P*<.05. Effect sizes were calculated using Cramer V, and adjusted standardized residuals with Bonferroni corrections (0.05 per number of comparisons) were used for post hoc tests in cases where the results were significant.

## Results

### Participant Sociodemographics

A total of 714 people completed the survey. The data were fairly evenly split between those collected from Brightside patients (368/714, 51.5%) and those collected from the general public (346/714, 48.5%). The combined sample was 67.1% (479/714) women; 73.1% (522/714) White, 7.3% (52/714) Asian, 6.7% (48/714) African American, and 7.4% (53/714) Hispanic or Latinx; largely aged 25 to 34 years (255/714, 35.7%) or 35 to 44 years (187/714, 26.2%); from either the Mid-Atlantic (131/714, 18.3%) or South Atlantic (129/714, 18.1%) regions of the country; and most (402/714, 56.3%) earning annual salaries of US $30,000 to US $100,000. Please refer to Table 1 for additional information about the participants, categorized by those not currently receiving any mental health treatment, those participating in psychotherapy treatment only, those participating in medication treatment only, and those engaging in both psychotherapy and medication treatments. The majority of the respondents (507/714, 71%) were receiving mental health treatment at the time of the survey.

**Table 1:** Sociodemographic characteristics of participants (N=714).

			No current mental health treatment (n=207), n (%)	Current psychotherapy treatment (n=40), n (%)			Current medication treatment (n=342), n (%)		Current combination treatment^a^ (n=125), n (%)		
**Gender**											
	Woman		100 (48.3)	23 (57.5)			268 (78.4)		88 (70.4)		
	Man		104 (50.2)	16 (40)			74 (21.6)		34 (27.2)		
	Nonbinary		1 (0.5)	0 (0)			0 (0)		2 (1.6)		
	Transgender		2 (1)	1 (2.5)			0 (0)		1 (0.8)		
**Race and ethnicity**											
	Black and African American		17 (8.2)	8 (20)			15 (4.4)		8 (6.4)		
	Hispanic or Latinx		18 (8.7)	7 (17.5)			15 (4.4)		13 (10.4)		
	White		137 (66.2)	11 (27.5)			285 (83.3)		89 (71.2)		
	Asian		25 (12.1)	10 (25)			10 (2.9)		7 (5.6)		
	Other^b^		10 (4.8)	4 (10)			17 (5)		8 (6.4)		
**Region**											
	New England		5 (2.4)	2 (5)			13 (3.8)		8 (6.4)		
	Mid-Atlantic		31 (15)	8 (20)			68 (19.9)		24 (19.2)		
	South Atlantic		42 (20.3)	7 (17.5)			53 (15.5)		27 (21.6)		
	Mountain		14 (6.8)	1 (2.5)			23 (6.7)		8 (6.4)		
	East North Central		27 (13)	7 (17.5)			51 (14.9)		8 (6.4)		
	East South Central		7 (3.4)	0 (0)			21 (6.1)		6 (4.8)		
	Pacific		34 (16.4)	6 (15)			55 (16.1)		19 (15.2)		
	West North Central		14 (6.8)	3 (7.5)			15 (4.4)		9 (7.2)		
	West South Central		22 (10.6)	4 (10)			41 (12)		14 (11.2)		
	Missing		11 (5.3)	2 (5)			2 (0.6)		2 (1.6)		
**Age (years)**											
	18-24		32 (15.5)		9 (22.5)		40 (11.7)			11 (8.8)	
	25-34		50 (24.2)		11 (27.5)		142 (41.5)			52 (41.6)	
	35-44	46 (22.2)				11 (27.5)		98 (21.7)			32 (25.6)
	45-54	17 (8.2)				7 (17.5)		32 (9.4)			21 (16.8)
	55-64	25 (12.1)				1 (2.5)		19 (5.6)			6 (4.8)
	65-74	27 (13)				1 (2.5)		10 (2.9)			2 (1.6)
	>75	10 (4.8)				0 (0)		1 (0.3)			1 (0.8)
**Annual income (US $)**											
	<30,000		36 (17.4)	4 (10)			83 (24.3)		25 (20)		
	30,000-100,000		127 (61.4)	21 (52.5)			191 (55.8)		63 (50.4)		
	>100,000		44 (21.2)	15 (37.5)			68 (19.9)		37 (29.6)		

^a^Psychotherapy and medication.

^b^*Other* included participants who chose Native American, Alaska Native, Pacific Islander, Middle Eastern, biracial, prefer not to say, or other.

### Other Results

**Figure 1:**
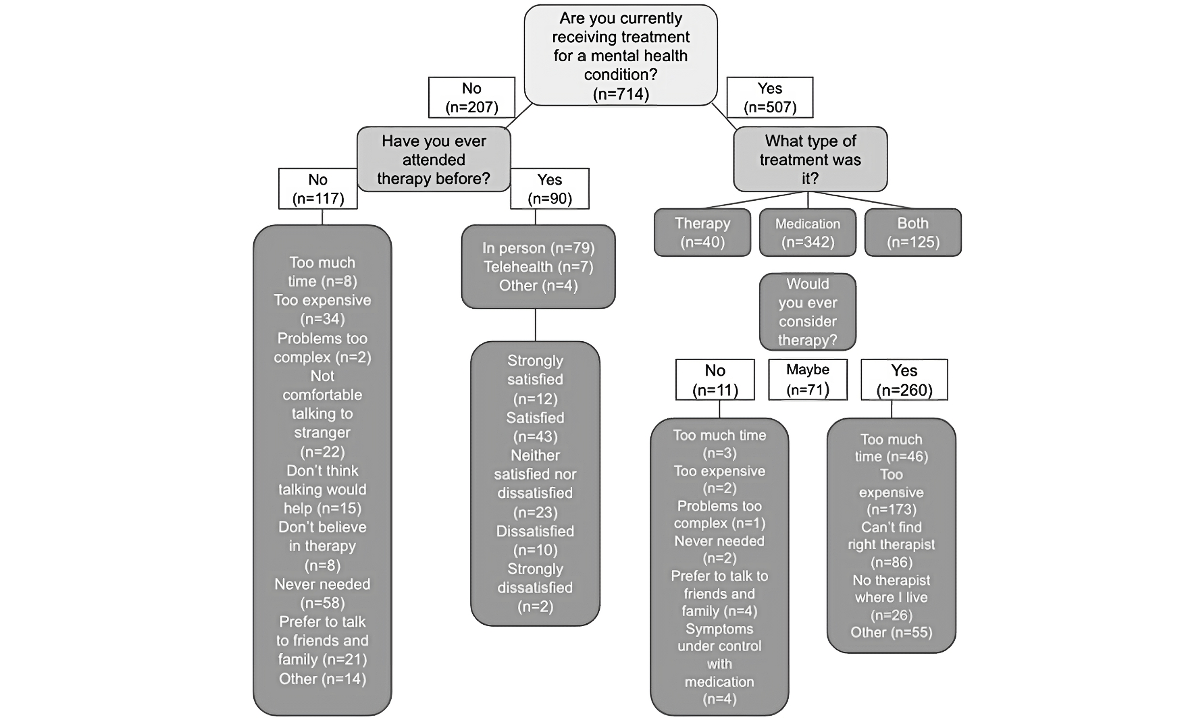
Responses to questions about willingness to engage in psychotherapy by those currently and not currently engaged in mental health treatment.

As can be seen in Figure 1, a slight majority of people (117/207, 56.5%) not currently in treatment have never participated in psychotherapy, although many had (90/207, 43.5%). The most frequently endorsed reasons for never having been in psychotherapy were as follows: never needed, too expensive, not comfortable talking to strangers, and preferring to talk to friends and family.

Most (55/90, 61%) were satisfied or strongly satisfied with their therapy services, although 26% (23/90) were neither satisfied nor dissatisfied, and 13% (12/90) were dissatisfied or strongly dissatisfied. There was no significant association between degree of satisfaction and group (Brightside patients vs general public: *χ*^2^_4_=5.6; *P*=.23), gender (*χ*^2^_12_=11.3; *P*=.51), race or ethnicity (*χ*^2^_16_=14.6; *P*=.93), age (*χ*^2^_24_=22.2; *P*=.57), income level (*χ*^2^_8_=10.6; *P*=.23), or region of the country (*χ*^2^_36_=40.1; *P*=.29). The vast majority of those in the public sample (315/346, 91%) had participated in psychotherapy in person, whereas for those in the Brightside sample, therapy was 100% internet based. Most of the respondents, if given a choice, would prefer internet-based psychotherapy (314/714, 44%), whereas 22.4% (160/714) prefer in-person psychotherapy, and 33.6% (240/714) report no preference. There were no significant differences in these preferences for therapy format by gender (*χ*^2^_6_=9.7; *P*=.05), race or ethnicity (*χ*^2^_8_=20.0; *P*=.13), age (*χ*^2^_12_=13.2; *P*=.21), or region of the country (*χ*^2^_18_=21.3; *P*=.27). There was a significant relationship between income level and stated therapy format preferences (*χ*^2^_4_=15.1; *P*<.01; Cramer V=0.14; *P*<.01). Post hoc tests revealed that those in the lowest (<US $30,000) income bracket were more likely to state that they had no preference, whereas those in the highest (>US $100,000) income bracket were significantly less likely to state that they had no preference (*P*=.005).

Of those currently in treatment (507/714, 71%), the majority (342/507, 67.5%) were using pharmacotherapy only, a minority (40/507, 7.9%) were engaged in psychotherapy only, and 24.7% (125/507) were engaged in both psychotherapy and pharmacotherapy (Figure 1). Of those engaged in pharmacotherapy only, the majority (260/342, 76%) would definitely consider psychotherapy, but it was perceived as too costly or difficult to find the right therapist, followed by *takes too much time*.

Of those not engaged in mental health treatment (207/714, 29%), the majority said that they might (98/207, 47.3%) or would (57/207, 27.5%) consider taking medication (not shown in Figure 1), and the primary reasons for not wanting to consider medication (52/207, 25.1%) were concerns about side effects (19/52, 37%), being against medications (16/52, 31%), believing that medications would not help (14/52, 27%), and being scared that medications would change one’s personality (12/52, 23%). In the same group (ie, those not currently engaged in treatment), a slight majority (117/207, 56.5%) had never engaged in psychotherapy. Of these, 49.6% (58/117) said that they never needed it, and 29.1% (34/117) cited cost as a deterrent, followed by being uncomfortable talking to strangers (22/117, 18.8%), preferring to talk to friends or family, (21/117, 17.9%), and disbelief that talking helps (15/117, 12.8%).

In the entire sample, more than half (484/714, 67.8%) had heard of *evidence-based therapy* such as CBT, whereas 32.2% (230/714) had not. A little more than half (414/714, 58%) thought that evidence-based therapy was *important* or *very important*, whereas approximately one-third (250/714, 35%) thought it neither important nor unimportant, and 7% (50/714) thought that it was *unimportant* or *very unimportant*. There was no significant association between views on evidence-based therapy and group (Brightside patients vs general public: *χ*^2^_8_=15.2; *P*=.06), gender (*χ*^2^_12_=14.7; *P*=.26), income (*χ*^2^_8_=8.0; *P*=.44), or region of the country (*χ*^2^_36_=25.9; *P*=.89). There was a significant association between opinions about evidence-based practice and race (*χ*^2^_16_=54.4; *P*<.01; Cramer V=0.17; *P*<.01). Post hoc tests revealed that those choosing the *other* racial or ethnicity category (*prefer not to say*) were more likely to say that evidence-based practice was completely unimportant (*P*<.001). There was also a significant association between opinions about evidence-based practice and age (*χ*^2^_24_=37.8; *P*<.05; Cramer V=0.14; *P*<.05). However, post hoc tests, after Bonferroni corrections, did not reveal any significant differences among the age groups (*P*>.001). The respondents were provided a definition of evidence-based practice and then again asked how important they thought it was. After being given this definition, 58.4% (417/714) of the entire sample said that it was *important* or *very important*, whereas approximately one-third again (243/714, 34%) thought it neither important nor unimportant, and 7.6% (54/714) thought that it was *unimportant* or *very unimportant*.

**Figure 2:**
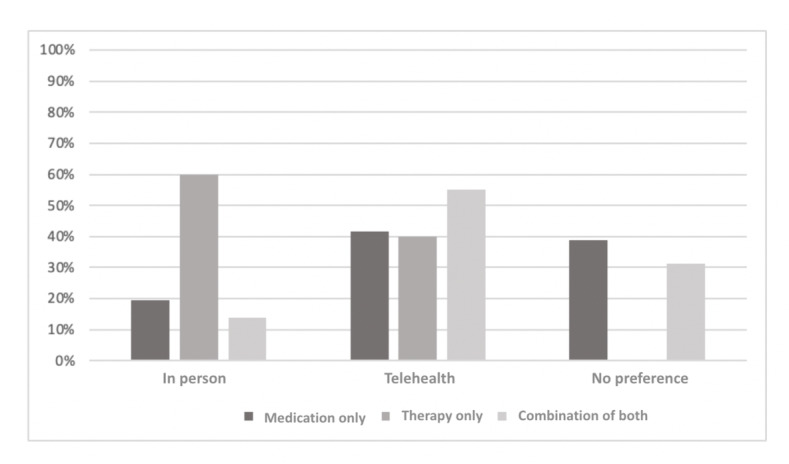
Participants’ views on the ideal location for therapy.

**Figure 3:**
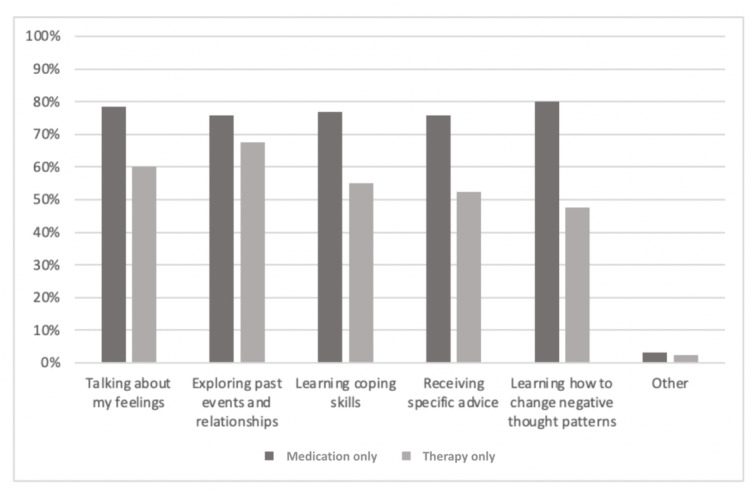
Participants’ responses to the question, "What do you expect therapy to include? (select all that apply)".

**Figure 4:**
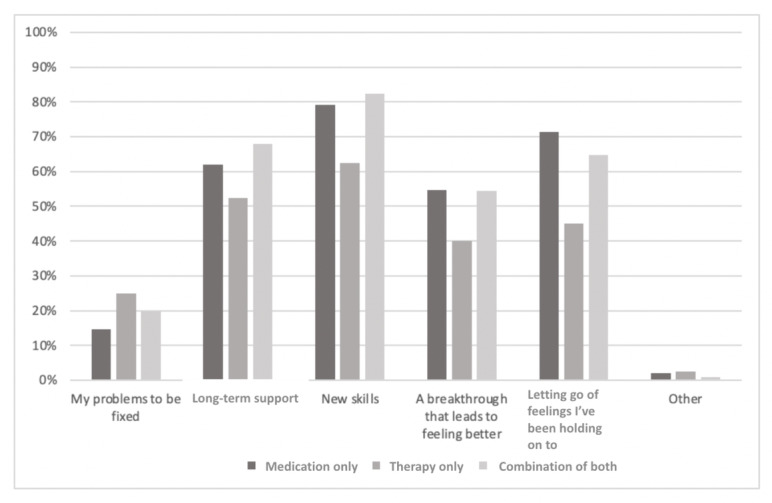
Participants’ responses to the question, "What do you expect to get out of therapy?".

The sample currently engaged in treatment was further categorized based on those currently engaged in pharmacotherapy only, psychotherapy only, or a combination of both. The results are presented graphically to highlight the differences in perceptions among the groups. The groups differed significantly in terms of whether they would prefer therapy to be internet based or in person (*χ*^2^_2_=47.8; *P*<.001; Cramer V=0.24; *P*<.001). As can be seen in Figure 2, those in the *therapy-only* group prefer *in-person* treatment to telehealth treatment to a far greater degree than those currently engaged in either of the medication groups (*P*=.006). In addition, those taking medication, whether alone or in combination with psychotherapy, were more likely to not have a strong preference regarding the *in-person* treatment versus telehealth treatment question (*P*=.006). Ideas about what psychotherapy consists of were largely similar across the groups (Figure 3), although the *medication-only* group was significantly more likely to expect therapy to include learning how to change negative thought patterns (*P*=.008). Expectations about what one might get out of therapy were largely similar across the groups (Figure 4), although the *therapy-only* group was less likely to expect therapy to include *letting go of feelings I’ve been holding on to* (*P*=.008).

**Figure 5:**
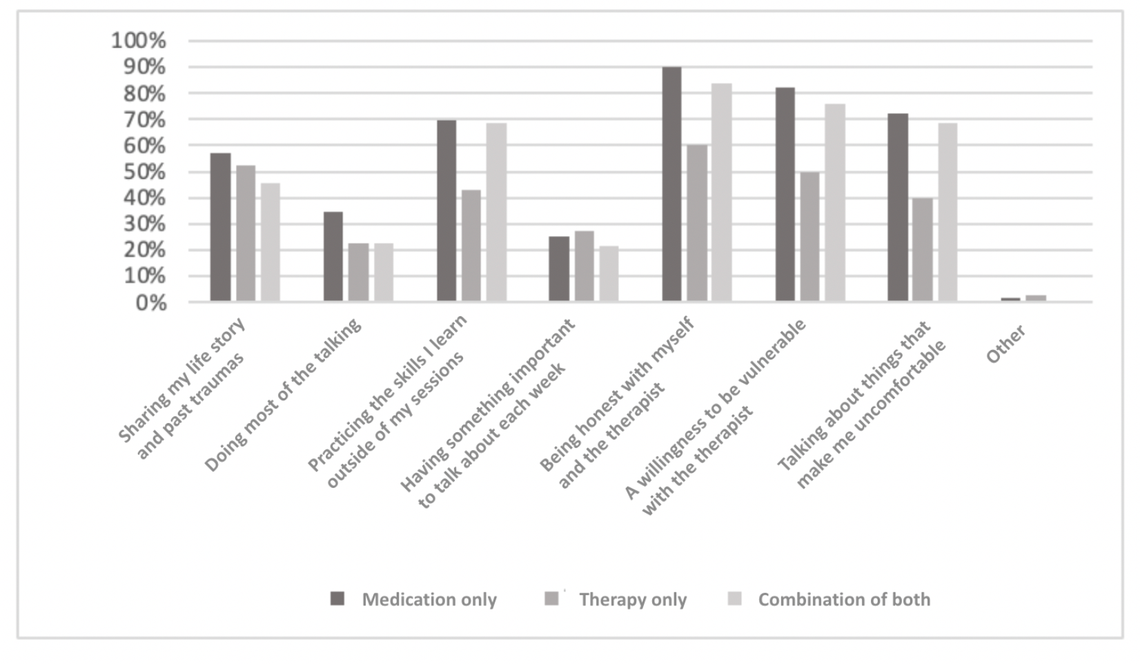
Participants’ responses to the question, "What do you think is expected of you in therapy? (select all that apply)".

Overall, *being honest with myself and the therapist* was the most frequently endorsed answer to the question *What do you think is expected of you in psychotherapy?* (615/714, 86.1%), followed by a willingness to be vulnerable (558/714, 78.1%). Across the groups (Figure 5), the *medication-only* group was significantly more likely to believe that *doing most of the talking* would be expected of them, as well as *being honest* and *a willingness to be vulnerable (P*=.008), and the *therapy-only* group was significantly less likely to endorse *practicing skills*, *being honest*, *a willingness to be vulnerable*, and *talking about things that make me uncomfortable* (*P*=.008).

**Figure 6:**
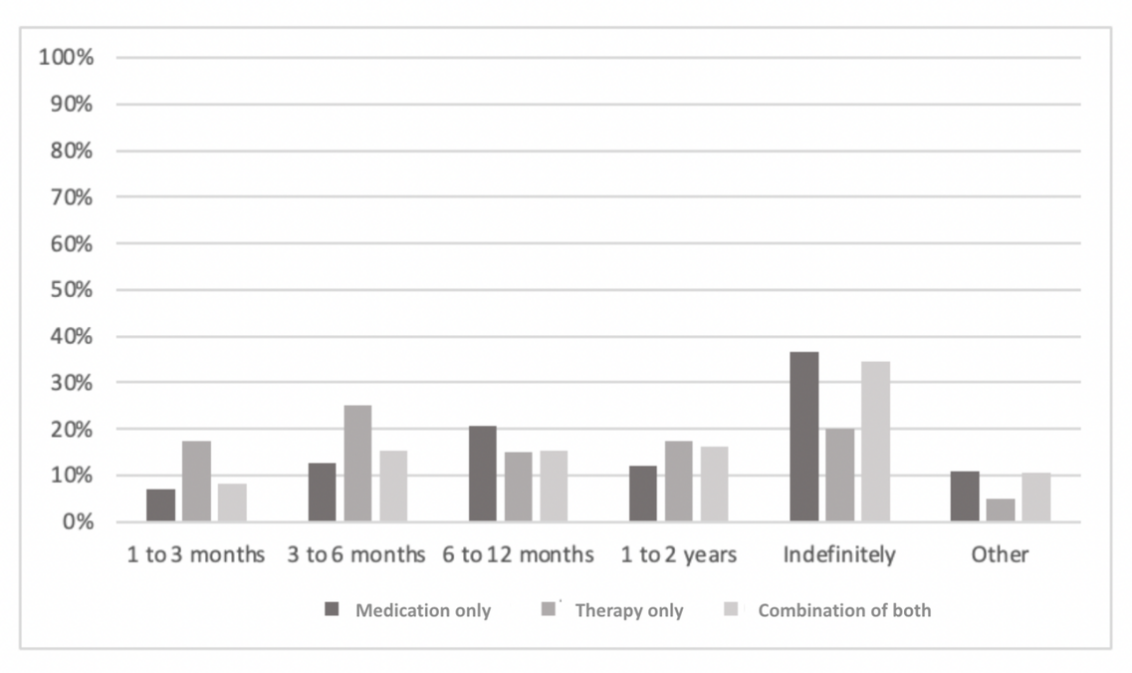
Participants’ responses to the question, "How long do you expect to be in therapy?".

Overall, the most commonly endorsed duration when asked *How long do you expect to be in therapy?* was *indefinitely* (250/714, 35%), followed by *6 to 12 months* (101/714, 14.1%). Very few (58/714. 8.1%) thought that therapy typically lasts 1 to 3 months. There was no significant difference among the groups (Figure 6; *χ*^2^_8_=16.5; *P*=.09; Cramer V=0.13; *P*=.09) on this variable.

**Figure 7:**
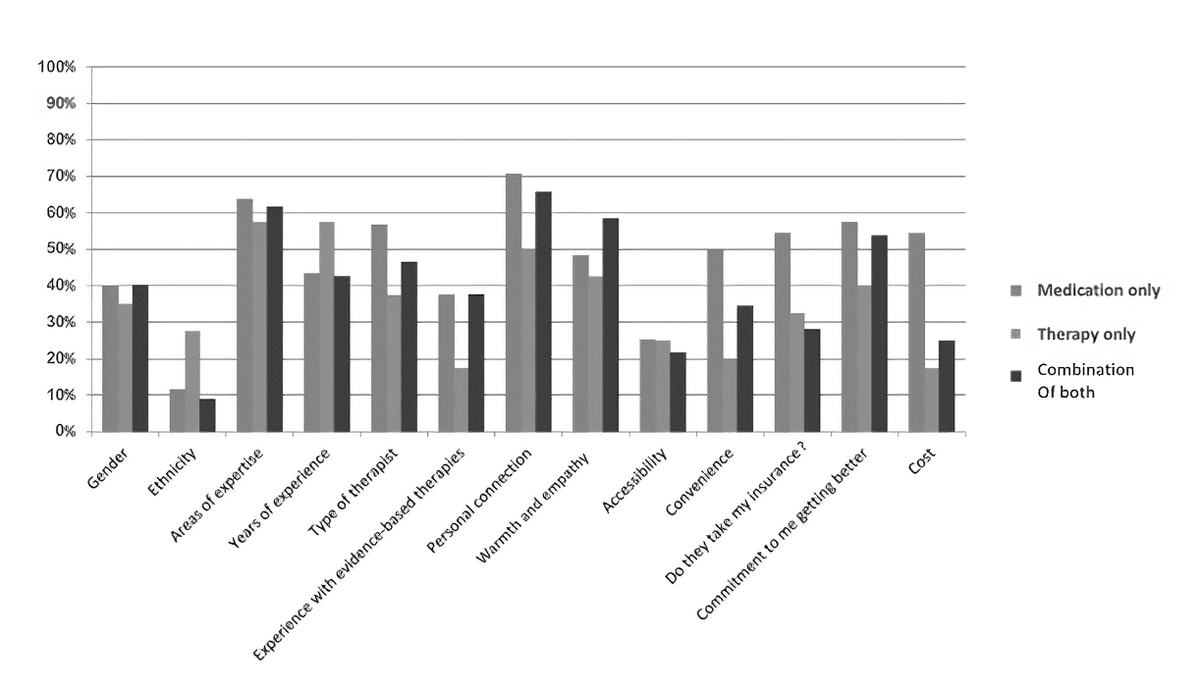
Participants’ responses to the question, "What factors do you consider when thinking about a good therapist fit?".

In response to a question about factors to consider when considering therapist compatibility, the most frequently endorsed factors within the entire sample were personal connection (483/714, 67.7%), areas of expertise (448/714, 62.7%), *their commitment to me getting better* (393/714, 55%), type of therapist (376/714, 52.7%), and warmth (359/714, 50.3%). Cost was endorsed by 44.2% (316/714), *whether they take my insurance* was endorsed by 46.2% (330/714), and expertise with evidence-based therapies was endorsed by 35.9% (256/714). Across the treatment subgroups (Figure 7), the *therapy-only* group was significantly more likely to endorse race or ethnicity as an important factor (*P*=.008). The *medication-only* group was more likely to endorse convenience, whereas the *therapy-only* group was less likely to endorse convenience (*P*=.008). The *medication-only* group was more likely to endorse *takes my insurance* and *cost* as important factors, whereas the *combination of medication and therapy* group was less likely to consider *takes my insurance* (*P*=.008).

**Figure 8:**
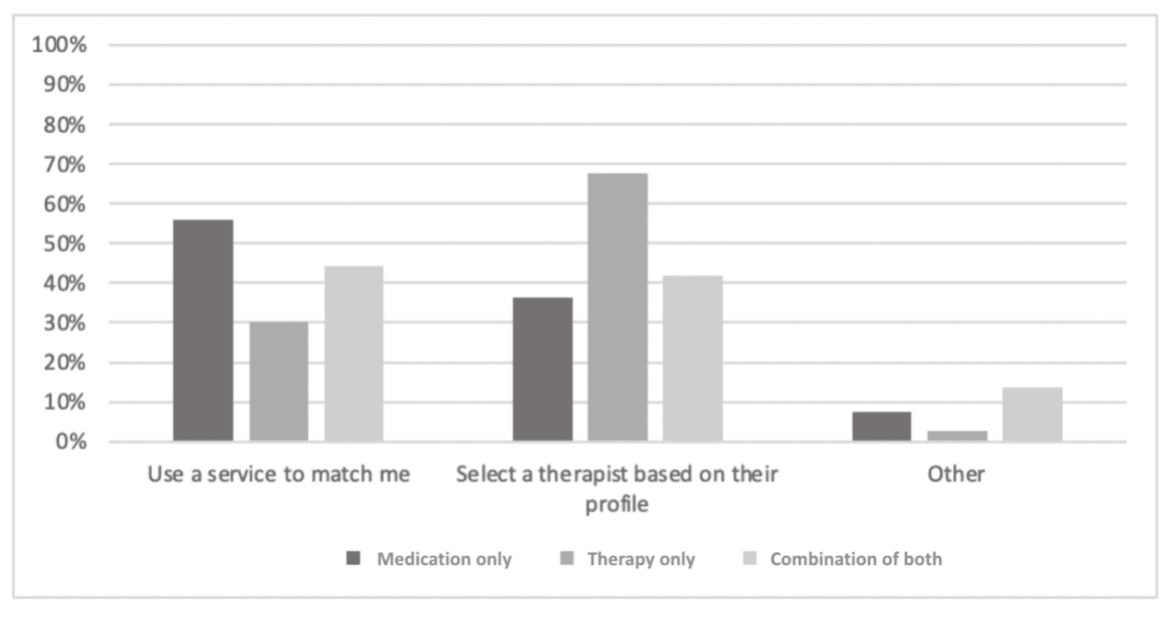
Participants’ responses to the question, "Would you rather use a service that matches you with a therapist based on preselected criteria, or would you rather choose your own therapist based on their profile?".

In the overall sample, a slim majority (366/714, 51.3%) preferred to use a matching service to select a therapist versus selecting one themselves based on the therapist’s profile (288/714, 40.3%). The groups differed significantly (Figure 8) on this question (*χ*^2^_4_=21.1; *P*<.001; Cramer V=0.14; *P*<.001). In the *medication-only* group, which was significantly more likely to prefer using a matching service (*P*<.005), 56.1% (192/342) would prefer to have a service match them to a therapist compared with 30% (12/40) of the *therapy-only* group and 44.8% (56/125) of the combined groups. Those in the *therapy-only* group clearly favored selecting their own therapist based on therapist profiles (27/40, 68%; *P*=.005).

**Figure 9:**
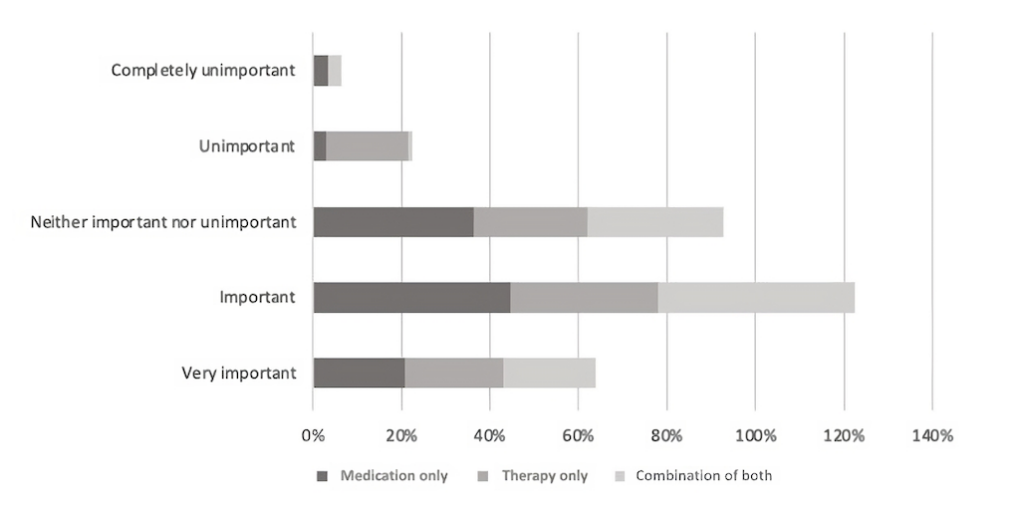
Participants’ responses to the question, "How important is it to you to receive evidence-based therapy?".

Finally, when specifically asked about the importance of receiving evidence-based care (Figure 9), the subgroups differed significantly in their opinions (*χ*^2^_8_=22.8; *P*<.004; Cramer V=0.17; *P*<.01). Post hoc tests revealed that those in the *therapy-only* group were more likely to say that evidenced-based therapy was *unimportant* (*P*<.003) at 18% (7/40) versus only 1.6% (2/125) in the *combination of medication and therapy* group and 2.9% (10/342) in the *medication-only* group.

## Discussion

### Principal Findings

This study seeks to provide initial data on public perceptions and attitudes around psychotherapy. The participants included current patients enrolled in care with Brightside, an internet-based national telemental health platform, as well as individuals from the general population. Specifically, the main aims of this study were to better understand (1) participant openness to, and satisfaction, with therapy; (2) perceptions, preferences, and expectations around therapy; and (3) perceptions of psychotropic medication. To our knowledge, this is the first survey to solicit perceptions of therapy from current and potential patients of internet-based mental health services. Originally created as a marketing assessment tool, the survey completed by participants investigated perceptions of the value in psychotherapy, why they pursue or do not pursue psychotherapy, and perceptions about psychotropic medications. There are several key takeaways from the survey.

Across all surveyed groups, there was a fairly even split regarding what individuals think is expected of them in therapy and what they expect to get out of therapy. The most striking finding was that a substantial number (250/714, 35%) of the people surveyed believe that psychotherapy lasts indefinitely. There were no significant differences among the subgroups, suggesting that these views are ubiquitous. In addition, among those engaged in pharmacotherapy only, the second most frequently indicated reason for not engaging in psychotherapy (despite the vast majority of total participants—221/342, 64.7%—having a positive view of it) was that it takes too much time. In fact, there is little relation between therapy duration and outcome [18,19]. The responsive regulation model suggests that, at least for routine care, therapy tends to end when gains reach a *good enough* level [20,21]. Empirically, in a systematic review of naturalistic studies worldwide, it was found that in most cases, therapy does not exceed 16 sessions [22]. In this survey, very few (58/714, 8.1%) believed that psychotherapy lasts 1 to 3 months, which is the most likely duration. There seems to be a misconception about the typical duration of therapy, which could be addressed with, for example, an educational campaign.

Going by the general population survey, 43.4% (150/346) of the respondents had never engaged in therapy. The most frequently endorsed reasons for never having been in psychotherapy were as follows: never needed, too expensive, not comfortable talking to strangers, and preferring to talk to friends or family. Of those engaged in pharmacotherapy only, the majority (260/342, 76%) would definitely consider psychotherapy, but it was perceived as too costly or difficult to find the right therapist, followed by *takes too much time*. Therefore, cost was a universal concern. These findings are interesting, given that those surveyed were likely contemplating internet-based therapy, which could potentially address at least some of the financial and accessibility concerns [17,23]. In this context, internet-based therapy refers to video telehealth interest as opposed to telephone only, given Brightside’s video-based care model. These findings are similar to those of surveys of primary care patients that suggest cost is a major perceived barrier to psychotherapy engagement [24,25]. Given the rising popularity of telehealth, it may be that telehealth addresses some of the structural barriers (such as cost and time). Furthermore, given our findings about participants perceiving therapy as lasting indefinitely, it is possible that cost concerns may be motivated by the perception that psychotherapy lasts indefinitely. Research has shown that predicting a patient’s necessary psychotherapy treatment duration is difficult even for clinicians themselves [26]. This uncertainty around treatment length may contribute to the perception that treatment goes on forever.

Of those who had engaged in psychotherapy and were not using Brightside’s internet-based platform, most (315/346, 91%) had attended in-person therapy. Overall, of those who engaged in therapy, most (55/90, 61%) were satisfied or strongly satisfied with it, whereas 26% (23/90) were neither satisfied nor dissatisfied. High degrees of satisfaction have been reported with specific psychotherapies [27,28] and with telehealth administration of therapy [29] more broadly, whereas data from this sample suggest that overall satisfaction with psychotherapy across these variables is largely favorable. Importantly, satisfaction did not vary according to demographic differences, suggesting that degree of satisfaction may not be dictated by demographic factors.

In the entire sample, more than half (484/714, 67.8%) had heard of *evidence-based therapy* such as CBT. Of those who had never heard of evidence-based care, a definition was offered, which did not seem to affect their *importance* ratings. Interestingly, the *therapy-only* group (7/40, 18%) was more likely to say that evidence-based care was *unimportant*. Prior survey research has indicated a clear preference for therapy guided by common factors, rather than by scientific evidence, among patients and nonpatients alike [10,13]. However, these surveys pitted scientific and relational characteristics against each other, creating a false dichotomy of exclusivity between the two. Another survey [14] posed questions differently, within the context of different problems, and also assessed therapists’ perceptions. The survey found that community members rated scientific credibility as important across problem types. Therapists substantially underestimated the importance of scientific characteristics to community members, particularly in the treatment of disorder-nonspecific issues. Therapists who valued research less in their own practice were more likely to underestimate the importance of scientific credibility to community members. This study suggests that most of the participants (414/714, 58%) thought that evidence-based practice was important. In addition, 36% (257/714) said that it was an important factor in selecting a therapist. However, counterintuitively, proving a definition did increase ratings of importance. It may be that more detailed education is needed (ie, explicitly detailing how such therapies might lead to better outcomes, rather than merely providing a definition) or that the definition used was insufficiently detailed (“evidence-based therapy is a type of therapy that has been shown to be effective in reducing psychological symptoms like depression and anxiety in scientific experiments”). In terms of preferences, it seems that attitudes about evidence-based care are neutral to positive, which is consistent with prior global research.

Not surprisingly, the vast majority of those in the public sample (315/346, 91%) had participated in psychotherapy in person, whereas for those in the Brightside sample, therapy was 100% internet based. Most of the respondents, if given a choice, would prefer internet-based psychotherapy (314/714, 44%), whereas 22.4% (160/714) prefer in-person therapy, and 33.6% (240/714) reported no preference. There were no differences in these preferences by demographics except income level such that those in the lowest and highest income brackets differed in terms of their likelihood for not having a preference one way or the other. For the subset of individuals currently in treatment, however, there were pertinent differences in preferences, with those currently engaged in psychotherapy preferring *in-person* treatment to telehealth treatment to a far greater degree than those currently engaged in either of the medication groups. In addition, those taking medication, whether alone or in combination with psychotherapy, were more likely to not have a strong preference regarding the in-person treatment versus telehealth treatment question.

Recent studies suggest good satisfaction with telehealth among those who have received it [30,31]. This again suggests the need for more public education focused on health. Telehealth, in terms of treating mental health disorders, has been empirically demonstrated to be equivalent [18-22,27,28,30-35] or superior to in-person treatment [36,37], with reduced no-show rates [35]. Certainly, the convenience of telehealth and potential cost savings may be attractive to many.

When looking at what is important to people as it relates to their therapist, several themes emerged across all surveyed groups. These included many of the *common factors* that have been empirically demonstrated to be paramount to positive clinical outcomes [38], including personal connection, good communication, and an open and honest relationship, as well as specific factors, such as therapist qualifications and confidence in their level of expertise. Cost was endorsed by 44.2% (316/714), *whether they take my insurance* was endorsed by 46.2% (330/714), and expertise with evidence-based therapies was endorsed by 35.9% (256/714). The *medication-only* group was much more likely to endorse *takes my insurance*, cost, and convenience, which points to a possible self-selection bias in terms of treatment selection.

The sample was fairly evenly split regarding whether they would prefer to use a matching service to select a therapist versus selecting one themselves based on the therapist’s profile. Although the *therapy-only* group clearly favored selecting their own therapist based on the therapist’s profile, the *medication-only* group was more likely to prefer using a matching service. Both these decisions have implications because patients who select their own therapist may perceive a greater locus of control in guiding their treatment-related decisions, whereas those who are matched with their therapist by the organization with whom they are working may conclude that effective algorithm-driven mechanisms ensure a good fit with their therapist, which will lead to better clinical outcomes. In either case, our findings suggest that patients are likely to perceive that regardless of how the assignment with their therapist occurred, they will attribute some of their therapeutic success to the well-fitting therapist selection when they perceive that therapy was effective and will blame some of their therapeutic failure on a poor initial therapist fit when they did not believe that therapy was helpful for their goals.

Finally, there was a largely positive reported attitude about medication. This is consistent with a trend of increasing acceptance of psychotropic medication [39], but the results were not statistically significant. Typically, those who are taking medication tend to have a more favorable view of it [40]. External locus of control predicts a decreased perception of personal agency regarding medication use and a greater likelihood of experiencing stigma related to medication use. Unlike in the case of therapy, the primary reason for participants not wanting to consider medication was not cost but rather fears about side effects, being generally opposed to taking medication, questioning medication efficacy, and worries that medication might change their personality. Mental health literacy, although not explored in this study, may also be a factor in attitudes toward medication [41]. Certainly, education surrounding the potential benefits of medication, as appropriate, may be warranted.

This study includes several limitations. Most notably, this study leverages a retrospective analysis of a survey originally designed for marketing purposes by a for-profit telemental health company. The generalizability of these results is limited because the participants constitute select convenience samples. Furthermore, Brightside indicated specific demographic parameters that limited the audience surveyed within the general population. Therefore, the responses are likely limited to a similar demographic in terms of generalizability. Furthermore, given the survey’s origin as a marketing tool, its psychometric properties are unknown. Specific numbers should be interpreted with the realization that future replication of findings is needed, and it should be noted that the most important information gleaned is represented by the major themes present in the data. Additional subanalyses were not completed to assess differences (beyond demographics) between the Brightside respondents and the general population, although these populations were combined for holistic analyses. The survey also queried *treatment expectations* over *outcome expectations* [42]. As such, questions about the perceived efficacy of telemental health, for example, should be included in future research.

### Conclusions

On the basis of the results of this survey study, several public health implications and future research directions can be surmised. Although generally favorable perceptions of both psychotherapy and medication were found, there may be a need for more in-depth education for patients and potential patients around what therapy is, how long it will tend to last, and scientific evidence surrounding its effectiveness. Education may also be necessary in the use of psychotropic medication because our findings suggest that the primary reason for not wanting to consider medication is not cost but fears about side effects and changes in personality. Public education may be key to greater participation in mental health treatment. Practitioners and those marketing their services might consider using their websites and marketing campaigns to counter some of the more common falsely held beliefs. Future research is needed to tease out any potential differences in perceptions between those who have completed telehealth treatment and those who have not. Future studies may also wish to explore locus of control as a factor in attitudes about medication, both among patients who are taking medication and those who are not.

## Appendix

Multimedia Appendix 1 Questions administered in the survey.

## Abbreviations

CBT: cognitive behavioral therapy
HIPAA: Health Insurance Portability and Accountability Act
IRB: institutional review board
UP: Unified Protocol

## Data Availability

The data sets generated and analyzed during this study are available from the corresponding author on reasonable request.
