# Supplementary material for: Consumer Expectations and Attitudes About Psychotherapy: Survey Study
Source: JMIR Form Res. 2023 Jun 8;7:e38696. doi: 10.2196/38696 (PMC10288349; doi:10.2196/38696)
Supplement: Multimedia Appendix 1 [file formative_v7i1e38696_app1.docx]

**Appendix A**

*Question 1 branching logic for the general population and Brightside patients solely receiving self-care support.*

**
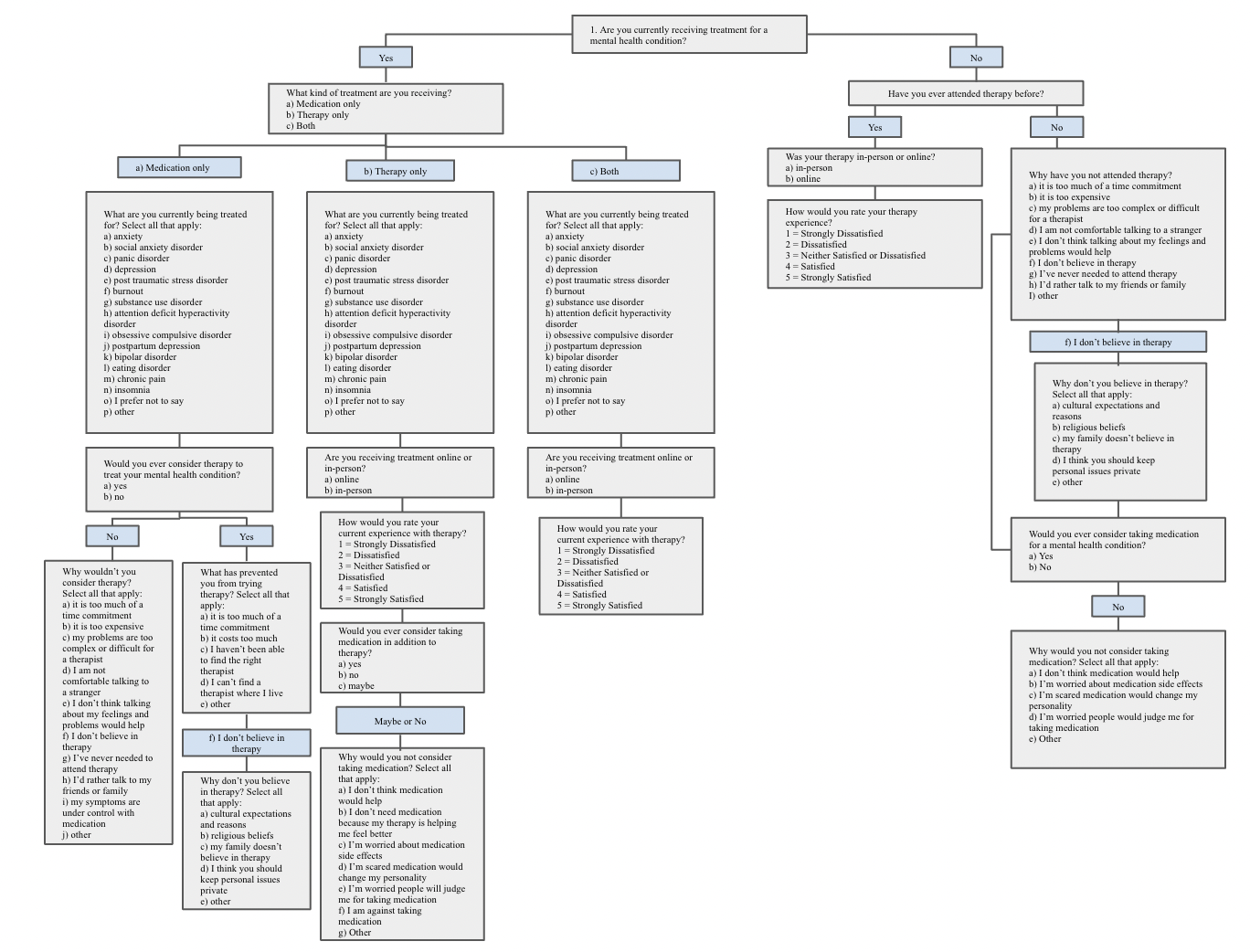
**

*Question 1 branching logic for Question 1 branching logic for. Question 1 branching logic for
Brightside patients solely Brightside patients solely Brightside patients receiving
receiving* *psychotherapy. receiving medication treatment. psychotherapy and medication treatment.*

*
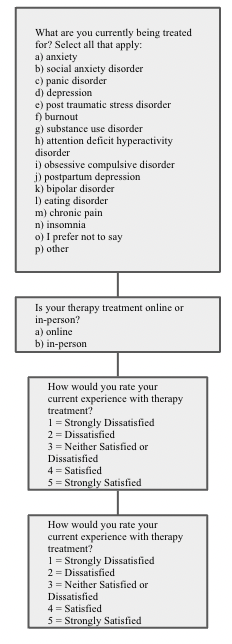

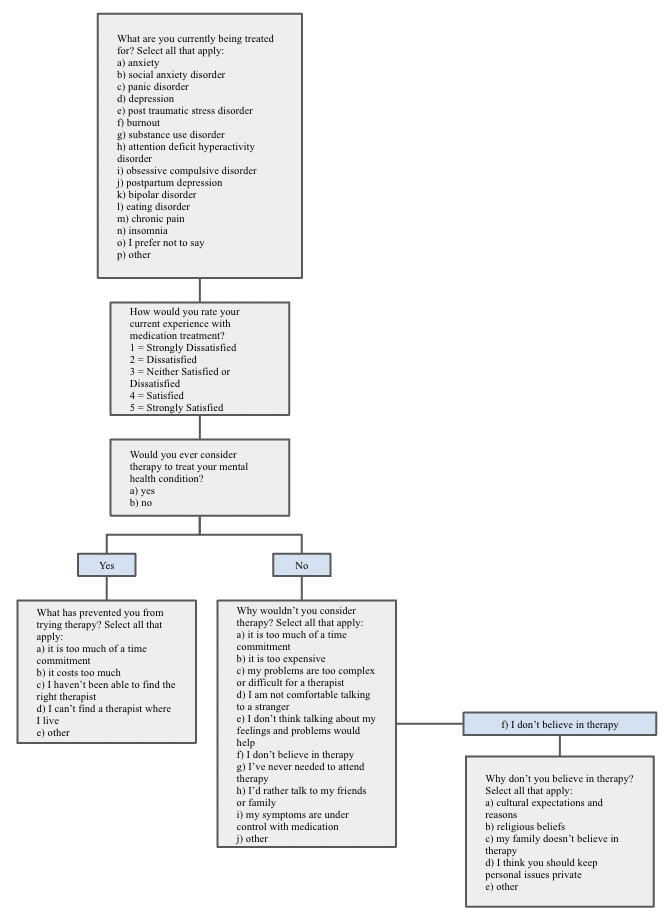
***
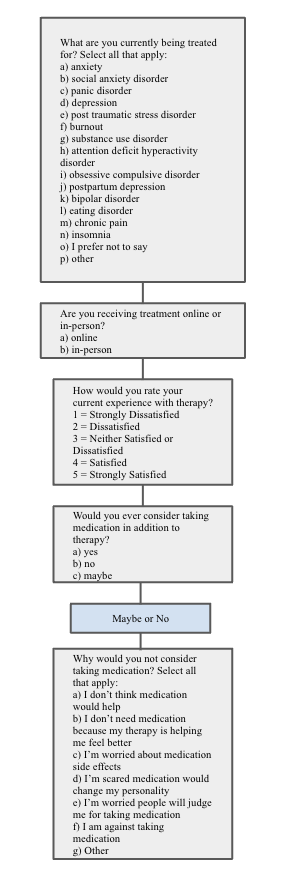
**

*
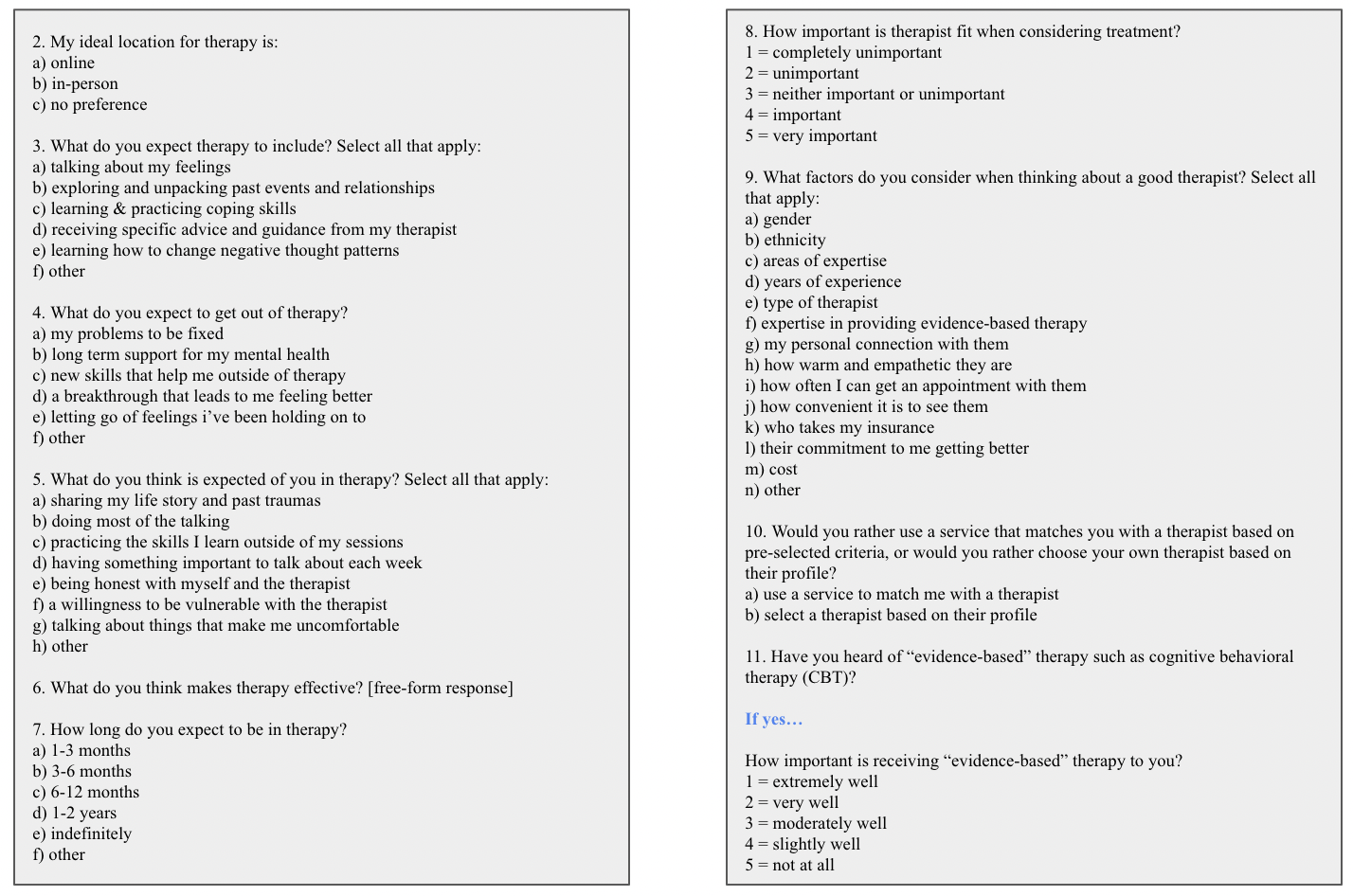
Survey questions 2-11, which all participants completed.*
